# Systematic identification of functional modules and cis-regulatory elements in *Arabidopsis thaliana*

**DOI:** 10.1186/1471-2105-12-S12-S2

**Published:** 2011-11-24

**Authors:** Jianhua Ruan, Joseph Perez, Brian Hernandez, Chengwei Lei, Garry Sunter, Valerie M Sponsel

**Affiliations:** 1 Department of Computer Science, The University of Texas at San Antonio, One UTSA Circle, San Antonio Texas 78249, USA; 2 Department of Biology, The University of Texas at San Antonio, One UTSA Circle, San Antonio Texas 78249, USA

## Abstract

**Background:**

Several large-scale gene co-expression networks have been constructed successfully for predicting gene functional modules and cis-regulatory elements in Arabidopsis (*Arabidopsis thaliana*)*.* However, these networks are usually constructed and analyzed in an *ad hoc* manner. In this study, we propose a completely parameter-free and systematic method for constructing gene co-expression networks and predicting functional modules as well as cis-regulatory elements.

**Results:**

Our novel method consists of an automated network construction algorithm, a parameter-free procedure to predict functional modules, and a strategy for finding known cis-regulatory elements that is suitable for consensus scanning without prior knowledge of the allowed extent of degeneracy of the motif. We apply the method to study a large collection of gene expression microarray data in Arabidopsis. We estimate that our co-expression network has ~94% of accuracy, and has topological properties similar to other biological networks, such as being scale-free and having a high clustering coefficient. Remarkably, among the ~300 predicted modules whose sizes are at least 20, 88% have at least one significantly enriched functions, including a few extremely significant ones (ribosome, *p* < 1E-300, photosynthetic membrane, *p* < 1.3E-137, proteasome complex, *p* < 5.9E-126). In addition, we are able to predict cis-regulatory elements for 66.7% of the modules, and the association between the enriched cis-regulatory elements and the enriched functional terms can often be confirmed by the literature. Overall, our results are much more significant than those reported by several previous studies on similar data sets. Finally, we utilize the co-expression network to dissect the promoters of 19 Arabidopsis genes involved in the metabolism and signaling of the important plant hormone gibberellin, and achieved promising results that reveal interesting insight into the biosynthesis and signaling of gibberellin.

**Conclusions:**

The results show that our method is highly effective in finding functional modules from real microarray data. Our application on Arabidopsis leads to the discovery of the largest number of annotated Arabidopsis functional modules in the literature. Given the high statistical significance of functional enrichment and the agreement between cis-regulatory and functional annotations, we believe our Arabidopsis gene modules can be used to predict the functions of unknown genes in Arabidopsis, and to understand the regulatory mechanisms of many genes.

## Background

Transcriptome analysis is the key of functional genomics research, and is often the hub of integrative analysis of -omics data. High-throughput expression profiling techniques such as DNA microarray [1] and RNA-seq [2] have resulted in thousands of gene expression data sets, each containing dozens to hundreds of experiments, being deposited into public databases such as the NCBI Gene Expression Omnibus (GEO) [3]. To effectively exploit this wealth of data, however, there is an urgent call for systematic methods to integrate data across multiple experiments.

Recently, there has been a surging interest in producing gene co-expression networks from microarray data, which have been shown as an important and useful technique in discovering knowledge from gene expression microarray data, with many interesting results being reported [4-18]. In a co-expression network, the nodes are genes and the edges indicate similar expression patterns between genes, according to some similarity metric. Co-expression is often correlated with functional relationships, such as physical interaction between the encoded proteins, or logical interaction in related biochemical and signaling pathways [19-22]. It has also been shown that gene co-expression networks have scale-free topology and community structure, similar to other biological networks [4,22]. Furthermore, it appears that genes with more co-expression links tend to be more evolutionarily conserved and essential [22]. Co-expression networks can be constructed in a number of ways, most of which involve some *ad hoc* parameters. The majority of the existing methods for constructing co-expression networks are based on some similarity threshold: two genes are connected by an edge whenever the similarity (or some transformation of it) between their expression levels is above a certain value [4,5,7-11,14-18,23]. This threshold is usually dataset dependent, although a few ideas have been proposed to help in the automatic selection of the threshold [5,9]. A problem with such threshold-based approachs is that different biological processes may show different levels of co-expression. Therefore, it is unlikely that a single threshold can be used to define all co-expression links. Recently, we and others proposed an asymmetric *k*-nearest-neighbor (aKNN)-based approach to construct gene co-expression networks [12,13,24,25]. Basically, for each gene *g*, we connect it to *k* other genes whose similarity to *g* is ranked the top *k* among all the genes. The advantage of this approach is that two genes sharing only weak expression similarity may be linked. We showed that a small *k* is needed to keep the whole network connected, and partitioning the network can result in higher module prediction accuracy than conventional clustering algorithms [24]. A problem with this approach, however, is that the microarray data needs to be preprocessed so that genes unrelated to the process of interest are removed before the construction of the network, to prevent them from being accidentally included in the network.

In this study, we propose a mutual k-nearest neighbor (mKNN) approach, which solves the problem of unspecific connections in the aKNN network, and is robust to random noise and scatter genes. We also propose a strategy to automatically determine the optimal *k* for constructing gene co-expression networks based on network topologies. We then apply a parameter-free modular discovery algorithm that we have developed previously [26] to partition the network into relatively dense subnetworks as candidates of functional modules. We also propose a cis-regulatory element finding algorithm that is suitable for consensus scanning without prior knowledge of the allowed extent of degeneracy of the motif. We applied the method to construct and analyze a whole-genome gene co-expression network for Arabidopsis (*Arabidopsis thaliana*) using more than one thousand microarray experiments. Prom the network we identified many interesting modules that are functionally coherent and potentially co-regulated. Remarkably, the functional modules we predicted are statistically much more significant than those reported by previous studies on similar data sets. In addition, we have predicted cis-regulatory elements for many of the functional modules, and the relationship between the cis-regulatory elements and the functional modules can often be confirmed by published results. Our results lead to the discovery of the largest number of Arabidopsis functional modules in the literature. Given the high statistical significance of Gene Ontology enrichment and the agreement between cis-regulatory and functional annotations of these genes modules, we believe that the results can be utilized to predict the functions of unknown genes in Arabidopsis, and to understand the regulatory mechanisms of many genes. As a proof of concept, we used the co-expression network to dissect the promoters of gibberellin metabolism and signaling genes, with some promising results that reveal new insight into the biosynthesis and signaling of the important plant hormone gibberellin.

## Results and discussion

We used a large collection of Arabidopsis gene expression microarray data that include 1388 microarrays for various growth conditions, developmental stages, and tissues of Arabidopsis [27,28]. The high quality of this collection of microarray data and diverse experimental conditions allow us to construct a global gene co-expression network that captures true functional relationship between pairs of genes.

### Overall network properties

Applying the automated network construction method, we obtained a gene co-expression network with *k* = 100, determined automatically by two topological measures (see Methods). The network contained 707602 edges, and a giant connected component (GCC) that included 21373 (~95%) of the 22591 genes assayed by the microarray. The next largest connected component had only 3 genes, while 1150 genes had no connections at all, which we omitted from further analysis. The mean and median vertex degree of the GCC is 33 and 26, respectively.

To compare, we randomized the gene expression data set and applied the same network construction method (with *k* = 100) to obtain a random network. It is important to note that this random network is not a random rewiring of the real network. In general, random rewiring would completely destroy the local modular structure of real networks, while a network constructed from randomized data would still have some modularity, because of the transitivity of most similarity measurement (i.e., *a* is close to *b* and *b* is close to c imply that *a* is close to c). We found that the real network and the random network had very different statistics. The largest connected component in the random network only contained 3183 genes, and 18187 genes has zero connections. Overall, this random network only contained 42354 edges; therefore, we estimated that our co-expression network had ~94% of accuracy.

**Figure 1:**
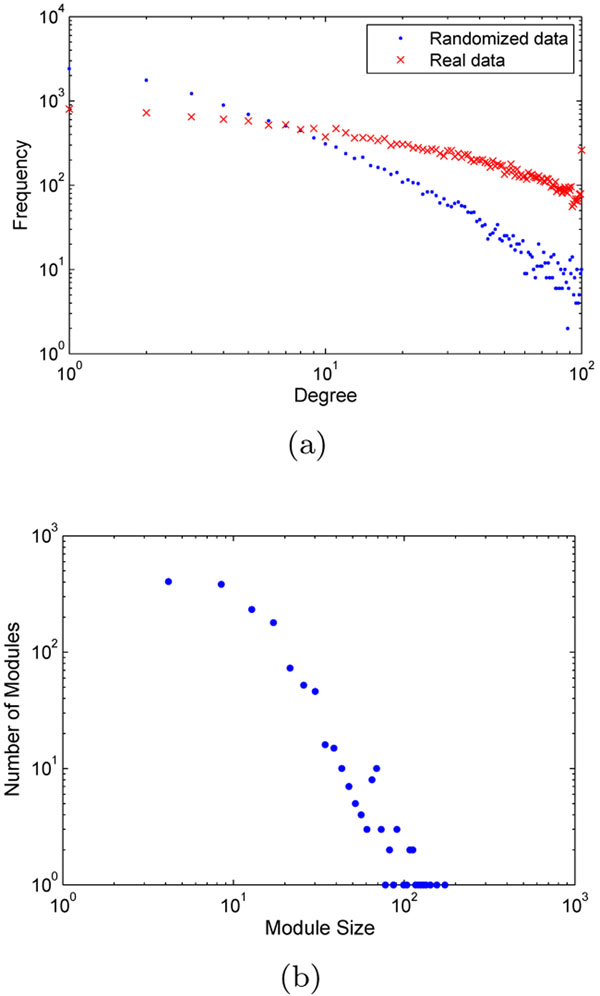
**Network properties.** (a) Degree distribution of the co-expression network constructed from real data or randomized data. (b) Module size distribution.

As shown in Figure 1(a), the real network seems to follow a power-law degree distribution, with an exponential truncation, which is common for many real-world networks. Note that the frequency of nodes with degree of 100 is artificially inflated, because the maximum degree allowed by the method is 100. The random network also appears to have a power-law degree distribution, but the network contains much fewer nodes of maximum degree. This shows that the requirement for two vertices to confirm their ranks eliminated most of the noise connections.

Furthermore, the real network has a much higher clustering coefficient than the random network (0.4001 vs. 0.1384). To show that the difference between the clustering coefficients of the two networks is not due to their sizes, we randomly sampled a subnetwork of size 3183 from the real network, and determined that its clustering coefficient is 0.399 ± 0.0199. Therefore, the real network has strong modularity that cannot be explained by the transitivity of the similarity measure. Also, after randomly rewiring, the clustering coefficients of both networks approaches zero.

Finally, it is worth mentioning that both the real network and the random network have similarly high degree correlations (0.6132 and 0.6084, respectively). This is different from asymmetric nearest neighbor networks [24], which typically has a negative degree correlation (data not shown). This may be an important property to consider when designing algorithms for analyzing these two types of networks.

### Enriched gene ontology terms

Using *HQcut*, we find 1473 modules from the largest component of the network, with sizes from 2 to 175. Many of them have small sizes. Module sizes follow a power-law distribution for the region between 10 and 100 (Figure 1(b)). Overall, there are ~800 modules of size at least 10, and ~300 modules of size at least 20. Gene Ontology analysis revealed that many of the modules have significantly enriched functions. Among the ~800 (300) modules whose sizes are at least 10 (20), 81.1% (88.0%) of them have at least one enriched function, with a Bonferroni corrected *p-*value < 0.05.

Table 1 shows 15 modules with the most significant Gene Ontology enrichment (top half), and 11 selected modules with significantly enriched cis-regulatory elements (bottom half), which will be discussed in the next subsection. The functional enrichment is extremely significant for some modules. For example, we have found several modules where the majority of genes are involved in the same specific functions (c1402, ribosome, *p* < 1E-300; c1473, photosynthetic membrane, *p* < 1.3E-137; c1051, proteasome complex, *p* < 5.9E-126). These modules also had statistically over-represented cis-regulatory elements, and the association between the functional modules and the cis-regulatory elements for many modules can be confirmed using previously published observations (see next subsection).

**Table 1:** Most significant modules according to function or motif

ID	Size	Enriched Function	*p-* value	Enriched Motif	*p-* value
c1402	174	structural constituent of ribosome	<1E-300	UP1ATMSD	<1E-16
c1473	110	photosynthetic membrane	1.30E-137	ACGTROOT1.1	4.00E-15
c1051	70	proteasome complex	5.90E-126	SITEIIATCYTC	6.00E-06
c1474	154	plastid	3.70E-93	UP1ATMSD	1.40E-08
c453	91	response to heat	1.20E-54	HSE	3.80E-11
c992	47	nucleosome assembly	1.20E-50	OCETYPEINTHISTONE	2.30E-14
c619	47	mitochondrion	5.40E-50	-	-
c1434	53	plastid	5.10E-41	UP1ATMSD	5.60E-05
c1463	56	chloroplast thylakoid	5.60E-38	-	-
c620	65	mitochondrion	1.00E-32	SITEIIATCYTC	1.60E-07
c1090	30	RNA splicing	1.70E-31	-	-
c991	45	DNA metabolic process	2.20E-31	E2FAT	1.10E-06
c148	112	nutrient reservoir activity	6.60E-31	RYREPEATBNNAPA	4.50E-12
c1257	55	endoplasmic reticulum	7.30E-30	UPRMOTIFIIAT	3.70E-11
c973	134	microtubule motor activity	8.20E-30	MYBCOREATCYCB1	6.10E-11
c701	17	aromatic compound metabolic process	8.00E-24	L1DCPAL1	5.90E-08
c294	26	response to water	2.60E-22	DRECRTCOREAT	5.10E-06
c778	99	circadian rhythm	2.00E-17	EVENINGAT	2.20E-16
c711	18	response to auxin stimulus	6.70E-15	MYCATRD22	4.90E-05
c488	72	defense response	7.10E-15	CGCGBOXAT	2.30E-10
c1369	59	ribonucleoprotein complex biogenesis and assembly	8.00E-15	UP2ATMSD	<1E-16
c489	81	response to abiotic stimulus	9.30E-09	CGCGBOXAT	<1E-16
c493	25	glutathione transferase activity	7.00E-07	OCSELEMENTAT.4	7.80E-16
c316	9	abscisic acid mediated signaling	1.90E-06	ABREATRD22	1.00E-06
c140	36	embryonic development ending in seed dormancy	1.60E-05	ABRERATCAL	4.00E-10
c302	14	response to abscisic acid stimulus	1.90E-04	ABRE3HVA1	9.20E-07

**Figure 2:**
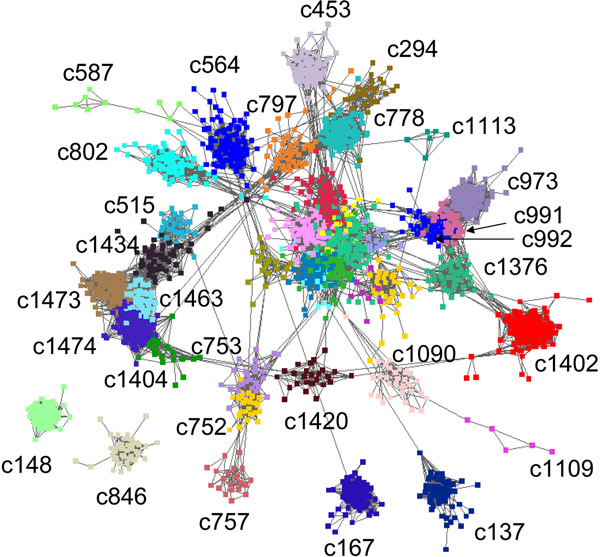
**Gene co-expression subnetwork of Arabidopsis.** Subnetwork contains genes in the top 40 functional modules with the highest statistical significance of enrichment of Gene Ontology terms.

Figure 2 shows a subnetwork that contains the top 40 modules with the highest statistical significance of enrichment of Gene Ontology terms. It is evident that most modules correspond to densely connected subnetworks. However, some of the modules appear to have close to linear structures, for example, c1109 (heat shock protein binding, *p* < 7E-19) and c587 (CCAAT-binding factor complex, *p* < 1E-22). This indicates that our method is able to identify not only densely connected functional modules, but also those sparsely connected, pathway-like structures. In addition, modules that are enriched with similar functions are often highly connected. For instance, c973, c991, and c992 are all involved in cell cycle, and c1473, c1474, c1434 and c1463 are involved in photosynthesis. These highly interconnected functional modules confirm a hierarchical organization of cellular functions [29].

### Enriched cis-regulatory elements

We annotated each module with a list of known cis-regulatory elements (motifs) from the PLACE database that are over-represented in the promoter sequences of the genes in the module (see Methods). Overall, 66.7% of the modules with size > 20 have at least one over-represented cis-regulatory element with a nominal *p-*value < 0.001.

Table 1 shows the most over-represented cis-regulatory element for each module. Note that statistical significance of the over-representation of cis-regulatory elements is typically much weaker than that of GO terms; this is because cis-regulatory elements are short and degenerate, and as a result may appear in promoter sequences simply by chance. Nevertheless, based on the information from the PLACE database [30], we find that many of the associations between the functional modules and the enriched cis-regulatory elements can be explained. For example, c453 is enriched with heat response genes, while the most significant motif in the module is a heat shock element [31]. Module c992 contains nucleosome assembly genes and is enriched with OCETYPEINTHISTONE, a composite motif known to be involved in regulating S phase-specific expression of a histone gene [32]. Module c991 has function in DNA replication, and is enriched with binding sites for the E2F family of transcription factors, which play a major role in regulating cell cycles [33]. Module c1257 contains genes associated with the endoplasmic reticulum (ER); the most significant motif in the module is UPRMOTIFIIAT, a cis-acting element regulating the unfolded protein response, which is activated in response to an accumulation of unfolded or misfolded proteins in the ER [34,35]. Another cell-cycle related module, c973, is enriched with MYBCOREATCYCB1, a core cis-regulatory element for the Arabidopsis cyclin B1:1 gene [36]. Module c701 is involved in aromatic compound metabolic process and is enriched with L1DCPAL1, a cis-regulatory element initially identified in a phenylalanine ammonia-lyase gene of Daucus carota (carrot) [37]. Module c294 contains water responsive genes and is enriched with a dehydration-responsive element, DRE/CRT [38,39]. For module c778, which is enriched with genes regulating circadian rhythm, the most significant motif in the module is EVENINGAT that is important for conferring rhythmicity to gene expression [40]. The OCSELEMENTAT motif enriched in c302 was found in the Arabidopsis glutathione S-transferase gene [41,42]. Finally, a few modules contain genes having functions in abiotic stress responses or embryonic development (c711, c488, c489, c316, c302, c140), while the corresponding cis-regulatory elements are either the well-known abscisic acid (ABA) responsive elements (ABREs) [43] or the ubiquitous CGCG-box, which is known to be involved in multiple signaling pathways in plants [44]. Interestingly, ABRE, UP1/2ATMSD, SITEIIATCYTC, and several other motifs have occurred in multiple modules, indicating that they may be involved in regulating multiple processes.

**Figure 3:**
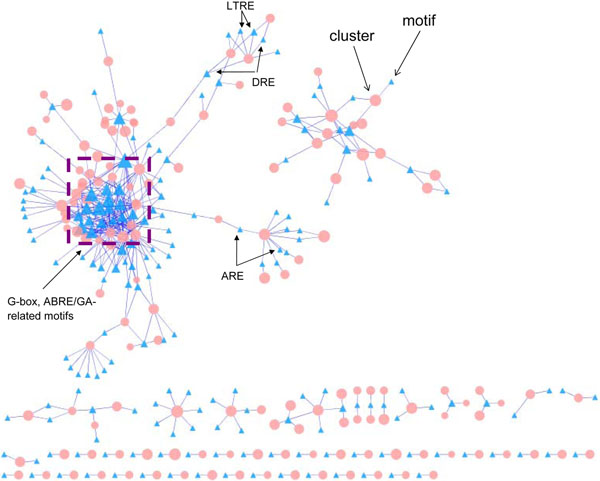
**Arabidopsis cis-regulatory network.** A circle represents a gene module. A triangle represents a motif. The size of a node is proportional to its module size or the number of modules it regulates.

Overall, a total of 177 unique motifs were found to be statistically significant in 144 modules, with a false discovery rate (FDR) ≤ 0.1. Figure 3 shows the final transcriptional regulatory network in Arabidopsis, where a circle represents a gene module, and a triangle represents a motif. The size of a node is proportional to its module size or number of modules it regulates. The motif clusters in the center of the left subnetwork contain many ABRE and gibberellin (GA)-related motifs, which is understandable as ABA and gibberellin regulate many biological processes in plants. Several other interesting cis-regulatory elements such as auxin response element (AREs), low temperature response elements (LTREs), and drought response elements (DREs) are loosely connected to the center, indicating the cross-talk among these stimulus response processes.

### Comparison with previous studies

Several previous studies have attempted to predict functional modules in Arabidopsis, using essentially the same microarray data compendium, based on co-expression networks or clustering methods [9,10,45,46]. It is worth noting that the previous co-expression networks were all constructed by some variants of the threshold-based methods (see Background). Remarkably, the enrichment of GO terms in our functional modules is much more significant than in all previous studies, to the best of our knowledge. For example, Horan et al. applied hierarchical clustering directly to the microarray data and obtained 916 clusters [45]. The most significant GO terms in their clusters are photosynthesis (*p* < 1.3E-89), ribosome (*p* < 5.3E-65), and proteasome complex (*p* < 1E-28). Mao et al. constructed a co-expression network using a Pearson correlation coefficient cutoff 0.75 [10]. Using the Markov clustering algorithm (MCL) [47], they identified 527 clusters. The five most significant clusters contain genes in photosynthesis (*p* < 1.4E-52), protein biosynthesis (*p* < 5.7E-52), DNA metabolism (*p* < 9.1E-52), starch metabolism (*p* < 3.2E-19), and response to heat (*p* < 1.7E-17).

Ma et al. [9] and Vandepoele et al. [46] have also used co-expression networks for predicting functional modules, but the overall goals/strategies of their studies are different from ours. Ma et al. attempted to find co-expressed neighbors of known guide genes. The five most significant GO terms found by Ma et al. are response to heat (*p* < 9.4E-55), chromatin (*p* < 7.5E-48), response to auxin (*p* < 3.6E-41), proteasome complex (*p* < 6.7E-29), and starch metabolism (*p* < 6.5E-18). The work of Vandepoele et al. combines co-expression with sequence-level conservation between Arabidopsis and poplar. The most significant GO terms they found are photosynthesis (*p* < 2.2E-87), ribosome biogenesis and assembly (6.1E-68), and DNA replication (*p* < 8.9E-26).

Finally it is worth noting that our network (mean vertex degree = 26) is much sparser than the network of Mao et al. (mean vertex degree = 165), and that of Vandepoele et al. (mean vertex degree = 717). Our network is more sparse, making it easier for analysis and visualization. At the same time, our network covers about 95% of the Arabidopsis genes, whereas the networks by Ma et al. and Mao et al. only cover about 30% of Arabidopsis genes. As a result, we are able to identify more functional modules than in these previous studies.

### Application: gene-centric analysis

**Figure 4:**
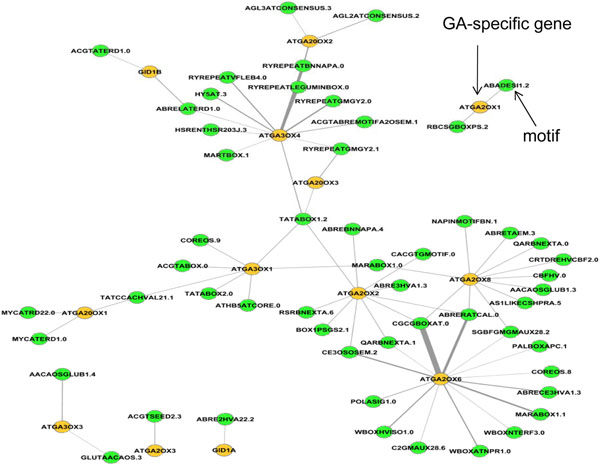
**Cis-regulatory network of Arabidopsis gibberellin metabolism and signaling genes.** Yellow and green nodes represent genes and cis-regulatory elements, respectively. The width of an edge is proportional to the significance of enrichment, measured by the negative logarithm of the *p-*value. The number after the dot following the motif name represents the number of mismatches allowed in order to obtain maximum statistical significance.

As an application, we used the co-expression network to study a set of gibberellin (GA) metabolism and signaling genes. The GAs are a group of plant hormones that singly or in combination with other hormones regulate many aspects of plant growth and development including germination, stem elongation and flowering [48]. We compiled 16 Arabidopsis genes that encode three small families of 2-oxoglutarate-dependent dioxygenase (2-ODD) enzymes in the GA metabolic pathway (4 GA 2-oxidases, 7 GA 3-oxidases and 5 GA 20-oxidases), and 3 genes that encode GA receptors (GID1a, GID1b, and GID1c). The amount of bioactive GA which binds to the receptor to transduce a biological response must be closely regulated by fine tuning GA biosynthesis and deactivation [48]. For each of the genes, we are interested in obtaining their cis-regulatory elements. We first obtained the co-expression neighbors for each gene. For each gene, we then combined itself and its co-expressed neighbors into a single list. We then searched for motifs that are not only over-represented in the list, but appeared in the promoter of the gene of interest (i.e., 2-ODD or GID1 genes). Figure 4 shows the network with the GA metabolism and signaling genes and their putative cis-regulatory elements. The width of an edge is proportional to the significance of enrichment. We predicted cis-regulatory elements for 11 out of 16 GA 2ODD genes and 2 out of 3 GA receptor genes. Abscisic acid (ABA) response elements (ABREs) play important roles in regulating several of these genes. It is well known that the balance between GA and ABA is an important factor regulating the development and growth of many plants [49]. It can also be seen that GA20ox and GA3ox families, both critical in the biosynthesis of bioactive GA, share more common cis-regulatory elements with each other than with members of the GA2ox family, which are responsible for GA deactivation [48]. The RYREPEATBNNAPA motif is also important for ABA responses [50].

## Conclusions

In this study, we have proposed a novel network-based method for identifying gene functional modules and cis-regulatory elements from a large microarray data set of Arabidopsis. The method included a mutual k-nearest-neighbor network construction method with automatic parameter selection, a modularity-based parameter-free module detection algorithm, and a cis-regulatory element finding algorithm that is suitable for consensus scanning without prior knowledge of the allowed extent of degeneracy of the motif. Since the method is completely parameter free, it is especially useful to be applied to analyzing microarray data sets that are of very large scale or are assaying poorly understood biological processes, where the appropriate network parameters and number of modules are difficult to estimate.

Applying our method to a large collection of Arabidopsis microarray data, we have significantly improved the prediction accuracy of functional modules compared to several previous studies. Our application leads to the discovery of the largest number of Arabidopsis functional modules in the literature; for many modules, we are able to annotate them with functional terms and cis-regulatory elements. Together, the high statistical significance of Gene Ontology enrichment and the agreement between cis-regulatory and functional annotations of these genes modules in Arabidopsis show that our Arabidopsis gene modules are excellent candidates of functional modules. Therefore, we believe that the results can be utilized to predict the functions of unknown genes in Arabidopsis, and to understand the regulatory mechanisms of many genes. As a proof of concept, we have used the co-expression network to dissect the promoters of gibberellin metabolism and signaling genes, with some promising results that reveal new insight into the biosynthesis and signaling of the important plant hormone gibberellin. We are constructing a database and web interface for querying the Arabidopsis gene co-expression network, the predicted functional modules and associated cis-regulatory elements.

## Methods

### Data

Gene expression microarray data were downloaded from The Arabidopsis Information Resource (TAIR) [51] and normalized according to the procedure of ATTED-II [52]. Promoter Sequences, defined as 1000bp upstream to transcription starting sites, were downloaded from TAIR. Known cis-regulatory elements were downloaded from the PLACE database [30].

### Network construction with topology-based parameter selection

We define a network as *G* = {*V*, *E*}, where *V* is the set of entities and *E* is the set of edges. Alternatively, we represent a network by its adjacency matrix, *W* = (*w_ij_*), where *w_ij_* = 1 if there is an edge between *v_i_* and *v_j_*, and 0 otherwise. Let *s_ij_* be the similarity between gene *i* and gene *j*, where similarity in this study is measured by Pearson correlation coefficient. With a given parameter *k*, the number of nearest neighbors to consider, the mutual *k*-nearest neighbor (mKNN)-based network is constructed by connecting any two genes that are within the top-*k* most similar genes of each other. That is, for gene *i* and gene *j* to be connected, they both need to be on the other gene’s top-*k* list. This is different from the previous aKNN-based method, where two genes are connected if one is on the top-*k* list of the other. Formally, we let *w_ij_* = 1 if *s_ij_* ≥ max{*s*_*ii*_*k*__, *s*_*jj*_*k*__} or 0 otherwise, where *i_k_* is the index of the gene whose similarity to gene *i* is smaller than exactly *k* – 1 other genes. In other words, |*x*, *x* ≠ *i* and *s_ix_* >*s*_*ii*_*k*__ | = *k* – 1.

**Figure 5:**
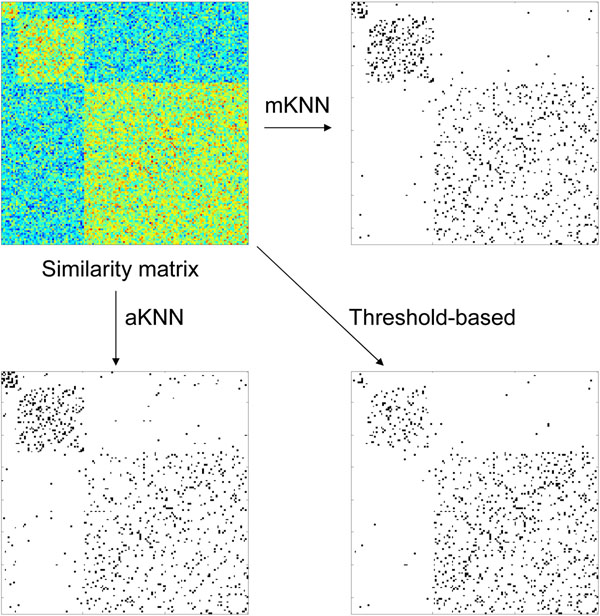
Illustration of three co-expression network construction methods.

The advantage of the mKNN methods compared to the threshold-based or aKNN-based methods (see Background) can be explained by a small example in Figure 5, which shows a similarity matrix containing three modules of different sizes (10, 40, and 100, respectively) and three networks constructed by the above methods. We have chosen parameters to make the three networks to have approximately the same density. Assume that the diagonal blocks (within-cluster gene pairs) in the similarity matrix are generated from the same distribution, and that the similarity scores in the off-diagonal regions (inter-cluster gene pairs) are generated from a different distribution. In the threshold-based method, all entries in the diagonal blocks have the same probability to be selected as edges. As a result, the expected edge density in different clusters will be the same. This, however, creates a huge disadvantage for the vertices in the smaller clusters, as they will have a smaller number of within-cluster edges compared to those in larger clusters.

Even worse, they may by chance have more inter-cluster edges. In the two KNN-based networks, in contrast, the smaller clusters usually have higher within-cluster edge densities, because the networks were constructed by connecting each vertex to the same number of neighboring vertices. (This does not mean all vertices have the same number of edges, however.) Indeed, as shown in Figure 5, the smallest cluster represented by the upper left diagonal block has much higher edge densities in the two KNN-based networks than in the threshold-based network. The mKNN-based network also has fewer inter-cluster edges in the off-diagonal regions than the aKNN-based network.

The parameter *k* is determined automatically based on the assumption that real networks have topological properties that are different from random networks [53]. For example, real networks often have a long-tail degree distribution, the small-world property, and high clustering coefficient [53]. Therefore, it is often suggested that these properties may be used to distinguish real networks from their random counterparts [5,53].

The topology-based parameter selection method works as follows. Given a co-expression network construction method and a topological measure Γ, we first decide a set of possible values for the parameter (i.e., *k*). We then construct a co-expression network using each parameter value, and compute the Γ value of the resulting network. At the same time, we also generate a random network by applying the same network construction method to a randomly permuted copy of the original expression data, and compute the corresponding Γ value of the random network. We then choose the network parameter that maximizes the difference between Γ_true_ and Γ_random_. Formally, let *G*(*A*, *k*) be the co-expression network generated on data set *A* using parameter *k*, and let *A^r^* be the permuted data, the optimal network G* is constructed as follows:(1)

Here we consider two types of topological measures. The first is the clustering coefficient, defined by the following formula: , where *N* is the number of vertices in the network, *d_i_* is the degree of vertex *i*, and *n_i_* is the number of connections between the neighbors of vertex *i.* In a recent study, Elo and colleagues recommended using clustering coefficient to choose the optimal network parameter [5]. Their experimental results were based exclusively on threshold-based networks.

Furthermore, a subtle but significant difference is that in their method, the random network was generated by randomly rewiring the true network. In contrast, in our method, the random network was generated by applying the same network construction method to a randomly permuted data set. As a randomly rewired network has no modular structure at all, its clustering coefficient is close to zero, when the network is sufficiently sparse. In contrast, the clustering coefficient of a network constructed from a random data set is non-negligible. In addition, our method searches for the parameter that corresponds to a global maximum value of Γ*_true_* – Γ*_random_*, while their method searches for the parameter that corresponds to the first local maximum of Γ*_true_* – Γ*_random_.* As a result, our method is less prone to noises than their method. The second type of topological measure we propose is a novel measurement specific for the mKNN method.

Assume that we choose parameter *k* in the mKNN method, and the average vertex degree of the resulting network is *n_k_*. We define the *normalized degree* of the network as *n_k_*/*k*. The normalized degree for any mKNN network is between 0 and 1. We use the normalized degree as the topological measure, and apply Equation (1) to choose a *k* that maximizes the difference between the normalized degree of the true network and that of its random counterpart. The rationale is as follows. In the mKNN network, the normalized degree is related to the conditional probability *p*(*s_ij_* ≥ *s*_*ii*_*k*__ | *s_ij_* ≥ *s*_*jj*_*k*__). Consider a similarity matrix where the similarity scores are completely random, which means *p*(*s_ij_* ≥ *s*_*ii*_*k*__) and *p*(*s_ij_* ≥ *s*_*jj*_*k*__) are independent. When each vertex chooses *k* neighbors, the probability for each of the *k* neighbors to also rank the current vertex as a top-*k* neighbor is exactly *k*/*N*, where *N* is the size of the network. The expected degree is therefore *k*^2^/*N* and the expected normalized degree would be *k*^2^/*N*/*k* = *k*/*N.* In a non-random similarity matrix that has modular structures, when *k* is small (or more precisely, smaller than a typical module size), the *k* nearest neighbors of most vertices are members of their modules, and therefore the expected degree for each vertex would be *k*^2^/*n*, where *n* is the size of the module that the vertex is in. The average degree of the network would be proportional to *ck*^2^/*N* where c is the number of modules. Consequently, the normalized degree would be proportional to *ck*/*N* and the difference between the normalized degree of the true network and that of the random network would grown as *k* grows, until *k* is about the same size of a typical module. After that, when *k* increases, new neighbors for most vertices would be chosen primarily from outside of their module, randomly. The probably *p*(*s_ij_* ≥ *s*_*ii*_*k*__ | *s_ij_* ≥ *s*_*jj*_*k*__) now drops to *k*/*N* from *k*/*n* and as a result, the difference between the normalized degree of the true and random networks would decrease when *k* increases.

### Module detection and annotation

Many module detection algorithms have been developed, most of which rely on some graph partitioning routines. We recently developed two graph partitioning algorithms within the framework of community discovery, which aims to identify the most interesting “natural” communities (i.e., relatively dense subnetworks) without user-tuned parameters [26]. The first algorithm, called *Qcut*, partitions a network by optimizing a well-known modularity function [26]. The second algorithm, called *HQcut*, solves the intrinsic resolution limit problem of the modularity function by iteratively calling *Qcut* to identify communities that does not contain any statistically significant sub-communities [26]. Here we employ the *HQcut* algorithm to the co-expression networks and treat the identified communities as candidates of functional modules. *HQcut* does not use any user-tunable parameters, except an optional statistical significance cutoff. We used a fixed cutoff (z-score = 2). Previously we have shown that in general the results of *HQcut* are not sensitive to this cutoff value [26].

We use enrichment of Gene Ontology terms to evaluate the significance of functional modules [54]. Specifically, given a gene subnetwork *s* and a Gene Ontology term *t*, the *p-*value for the enrichment of *t* in *s* is estimated by the cumulative hypergeometric test:(2)

where *N* is the number of genes in the genome, m is the size of the subnetwork, *n* is the number of genes in the genome with function *t*, and *a* is the number of genes in *s* with function *t.*

### Discovery of cis-regulatory elements and construction of cis-regulatory network

To establish the connection between co-expression and co-regulation in Arabidopsis microarray data, we explore the known transcription factor binding sites to find cis-regulatory elements (motifs) within each functional module. To do this, the promoter region (1000 bp upstream from the transcription start site) of each gene in a module is scanned with over 500 known motifs curated in the PLACE database, represented as consensus sequences [30]. The idea is that if a motif is found to be enriched in the genes’ promoters in a module, then perhaps those genes are regulated by that motif. To account for motif degeneracy, we allow a certain number of mismatches during the motif scanning. For long consensus sequences, this is necessary because many transcription factor binding sites are different from their canonical consensus sequences.

How to determine the number of mismatches to be allowed, however, is not trivial. We propose a simple strategy to search the optimal number of mismatches (for each motif) that can result in the most significant enrichment of the motif in a particular module. The sequence occurrence of the motif (with up to *l* mismatches) within a module is compared to that in the entire genome and the enrichment of the motif in the module is computed using the cumulative hyper-geometric test similarly as for testing the enrichment of Gene Ontology terms using Equation (2), where *t* now means a motif rather than a functional term. We vary *l* between 0 and *L*, where *L* is proportional to the length of the motif, and choose the optimal *l* that gives the most significant enrichment of the motif.

The final cis-regulatory network is constructed by treating functional modules and motifs as vertices, and an edge is created between a module and a motif if the motif is determined to be significantly enriched in the module. Since we are testing thousands of motif candidates (with different number of mismatches), *p-*values must be corrected for multiple hypothesis testing problem. We therefore computed the false discovery rate (FDR) using Benjamini’s and Hochberg’s procedure [55]. A motif is considered significantly enriched in a module if the FDR is no greater than 10%.

### Gene-centric co-expression and cis-regulatory element analysis

For each GA 2-oxoglutarate-dependent dioxygenase (2-ODD) gene or GA receptor (GID) gene, we first get its neighboring genes in the co-expression network; we then apply motif analysis to find motifs that are not only present in the 2ODD gene promoter, but also over-represented in its neighboring gene promoters, where over-representation is determined using Equation (2). We used a *p-*value cutoff 0.001. Such motifs are putative cis-regulatory elements for the 2ODD or GID gene.

## Competing interests

The authors declare that they have no competing interests.

## Authors’ contributions

JR and VMS established the research collaboration and coordinated the project. VMS and GS provided the necessary biological background to the project. JR designed and implemented the algorithms. JP, BH and JR carried out the analysis of Arabidopsis expression data. JP, BH and CL performed the motif analysis. VMS, GS, and JR interpreted the biological results. JR wrote the paper and VMS and GS helped with the manuscript preparation. All authors read and approved the final manuscript.

## References

[B1] Schena M Shalon D Davis RW Brown PO Quantitative monitoring of gene expression patterns with a complementary DNA microarray Science 1995 270 467 470 10.1126/science.270.5235.467 7569999

[B2] Wang Z Gerstein M Snyder M RNA-Seq: a revolutionary tool for transcriptomics Nat Rev Genet 2009 10 57 63 10.1038/nrg2484 19015660 PMC2949280

[B3] Barrett T Troup DB Wilhite SE Ledoux P Rudnev D Evangelista C Kim IF Soboleva A Tomashevsky M Edgar R NCBI GEO: mining tens of millions of expression profiles—database and tools update Nucleic Acids Res 2007 35 Database issue 760 765 http://www.hubmed.org/display.cgi?uids=17099226 10.1093/nar/gkl887 PMC1669752 17099226

[B4] Carter S Brechbühler C Griffin M Bond AT Gene co-expression network topology provides a framework for molecular characterization of cellular state Bioinformatics 2004 20 2242 2050 10.1093/bioinformatics/bth234 15130938

[B5] Elo L Järvenpää H Oresic M Lahesmaa R Aittokallio T Systematic construction of gene coexpression networks with applications to human T helper cell differentiation process Bioinformatics 2007 23 2096 103 10.1093/bioinformatics/btm309 17553854

[B6] Faith JJ Hayete B Thaden JT Mogno I Wierzbowski J Cottarel G Kasif S Collins JJ Gardner TS Large-scale mapping and validation of Escherichia coli transcriptional regulation from a compendium of expression profiles PLoS Biol 2007 5 e8 10.1371/journal.pbio.0050008 17214507 PMC1764438

[B7] Ghazalpour A Doss S Zhang B Wang S Plaisier C Castellanos R Brozell A Schadt EE Drake TA Lusis AJ Horvath S Integrating genetic and network analysis to characterize genes related to mouse weight PLoS Genet 2006 2 e130 10.1371/journal.pgen.0020130 16934000 PMC1550283

[B8] Lee HK Hsu AK Sajdak J Qin J Pavlidis P Coexpression analysis of human genes across many microarray data sets Genome Res 2004 14 1085 1094 10.1101/gr.1910904 15173114 PMC419787

[B9] Ma S Gong Q Bohnert HJ An Arabidopsis gene network based on the graphical Gaussian model Genome Res 2007 17 1614 1625 10.1101/gr.6911207 17921353 PMC2045144

[B10] Mao L Van Hemert JL Dash S Dickerson JA Arabidopsis gene co-expression network and its functional modules BMC Bioinformatics 2009 10 346 10.1186/1471-2105-10-346 19845953 PMC2772859

[B11] Oldham MC Horvath S Geschwind DH Conservation and evolution of gene coexpression networks in human and chimpanzee brains Proc Natl Acad Sci USA 2006 103 17973 17978 10.1073/pnas.0605938103 17101986 PMC1693857

[B12] Ray M Ruan J Zhang W Variations in the transcriptome of Alzheimer’s disease reveal modular networks involved in cardiovascular diseases Genome Biol 2008 9 R148 10.1186/gb-2008-9-10-r148 18842138 PMC2760875

[B13] Stuart JM Segal E Koller D Kim SK A gene-coexpression network for global discovery of conserved genetic modules Science 2003 302 249 255 10.1126/science.1087447 12934013

[B14] Tsaparas P Mariño-Ramírez L Bodenreider O Koonin EV Jordan IK Global similarity and local divergence in human and mouse gene co-expression networks BMC Evol Biol 2006 6 70 10.1186/1471-2148-6-70 16968540 PMC1601971

[B15] van Noort V Snel B Huynen MA The yeast coexpression network has a small-world, scale-free architecture and can be explained by a simple model EMBO Rep 2004 5 280 284 10.1038/sj.embor.7400090 14968131 PMC1299002

[B16] Weston DJ Gunter LE Rogers A Wullschleger SD Connecting genes, coexpression modules, and molecular signatures to environmental stress phenotypes in plants BMC Syst Biol 2008 2 16 10.1186/1752-0509-2-16 18248680 PMC2277374

[B17] Zhou X Kao MC Wong WH Transitive functional annotation by shortest-path analysis of gene expression data Proc Natl Acad Sci USA 2002 99 12783 12788 10.1073/pnas.192159399 12196633 PMC130537

[B18] Zhu D Hero AO Cheng H Khanna R Swaroop A Network constrained clustering for gene microarray data Bioinformatics 2005 21 4014 4020 10.1093/bioinformatics/bti655 16141248

[B19] Ge H Liu Z Church GM Vidal M Correlation between transcriptome and interactome mapping data from Saccharomyces cerevisiae Nat Genet 2001 29 482 486 http://www.hubmed.org/display.cgi?uids=11694880 10.1038/ng776 11694880

[B20] Jansen R Greenbaum D Gerstein M Relating whole-genome expression data with protein-protein interactions Genome Res 2002 12 37 46 http://www.hubmed.org/display.cgi?uids=11779829 10.1101/gr.205602 11779829 PMC155252

[B21] Kemmeren P van Berkum NL Vilo J Bijma T Donders R Brazma A Holstege FC Protein interaction verification and functional annotation by integrated analysis of genome-scale data Mol Cell 2002 9 1133 1143 http://www.hubmed.org/display.cgi?uids=12049748 10.1016/S1097-2765(02)00531-2 12049748

[B22] Jordan IK Mariño-Ramírez L Wolf YI Koonin EV Conservation and coevolution in the scale-free human gene coexpression network Mol Biol Evol 2004 21 2058 2070 10.1093/molbev/msh222 15282333

[B23] Magwene PM Kim J Estimating genomic coexpression networks using first-order conditional independence Genome Biol 2004 5 R100 10.1186/gb-2004-5-12-r100 15575966 PMC545795

[B24] Ruan J Dean AK Zhang W A general co-expression network-based approach to gene expression analysis: comparison and applications BMC Syst Biol 2010 4 8 10.1186/1752-0509-4-8 20122284 PMC2829495

[B25] Aggarwal A Guo DL Hoshida Y Yuen ST Chu KM So S Boussioutas A Chen X Bowtell D Aburatani H Leung S Tan P Topological and functional discovery in a gene coexpression meta-network of gastric cancer Cancer Res 2006 66 232 241 10.1158/0008-5472.CAN-05-2232 16397236

[B26] Ruan J Zhang W Identifying network community structures with a high resolution Phys Rev E Stat Nonlin Soft Matter Phys 2008 77 016104 18351912 10.1103/PhysRevE.77.016104

[B27] Schmid M Davison TS Henz SR Pape UJ Demar M Vingron M Schölkopf B Weigel D Lohmann JU A gene expression map of Arabidopsis development Nat Genet 2005 37 501 506 10.1038/ng1543 15806101

[B28] Kilian J Whitehead D Horak J Wanke D Weinl S Batistic O D'Angelo C Bornberg-Bauer E Kudla J Harter K The AtGenExpress global stress expression data set: protocols, evaluation and model data analysis of UV-B light, drought and cold stress responses Plant J 2007 50 347 363 10.1111/j.1365-313X.2007.03052.x 17376166

[B29] Rives AW Galitski T Modular organization of cellular networks Proc Natl Acad Sci USA 2003 100 1128 1133 10.1073/pnas.0237338100 12538875 PMC298738

[B30] Higo K Ugawa Y Iwamoto M Korenaga T Plant cis-acting regulatory DNA elements (PLACE) database:1999 Nucleic Acids Res 1999 27 297 300 10.1093/nar/27.1.297 9847208 PMC148163

[B31] Bharti K Schmidt E Lyck R Heerklotz D Bublak D Scharf K Isolation and characterization of HsfA3, a new heat stress transcription factor of Lycopersicon peruvianum Plant J 2000 22 355 65 10.1046/j.1365-313x.2000.00746.x 10849352

[B32] Taoka K Kaya H Nakayama T Araki T Meshi T Iwabuchi M Identification of three kinds of mutually related composite elements conferring S phase-specific transcriptional activation Plant J 1999 18 611 23 10.1046/j.1365-313x.1999.00486.x 10417712

[B33] Mariconti L Pellegrini B Cantoni R Stevens R Bergounioux C Cella R Albani D The E2F family of transcription factors from Arabidopsis thaliana. Novel and conserved components of the retinoblastoma/E2F pathway in plants J Biol Chew 2002 277 9911 9 10.1074/jbc.M110616200 11786543

[B34] Schröder M Kaufman R The mammalian unfolded protein response Annu Rev Biochem 2005 74 739 789 10.1146/annurev.biochem.73.011303.074134 15952902

[B35] Martinez I Chrispeels M Genomic analysis of the unfolded protein response in Arabidopsis shows its connection to important cellular processes Plant Cell 2003 15 561 76 10.1105/tpc.007609 12566592 PMC141221

[B36] Planchais S Perennes C Glab N Mironov V Inze D Bergounioux C Characterization of cis-acting element involved in cell cycle phase-independent activation of Arath;CycB1;1 transcription and identification of putative regulatory proteins Plant Mol Biol 2002 50 111 27 12139003 10.1023/a:1016018711532

[B37] Maeda K Kimura S Demura T Takeda J Ozeki Y DcMYB1 acts as a transcriptional activator of the carrot phenylalanine ammonia-lyase gene (DcPAL1) in response to elicitor treatment, UV-B irradiation and the dilution effect Plant Mol Biol 2005 59 739 52 10.1007/s11103-005-0910-6 16270227

[B38] Yamaguchi-Shinozaki K Shinozaki K A novel cis-acting element in an Arabidopsis gene is involved in responsiveness to drought, low-temperature, or high-salt stress Plant Cell 1994 6 251 64 8148648 10.1105/tpc.6.2.251 PMC160431

[B39] Stockinger E Gilmour S Thomashow M Arabidopsis thaliana CBF1 encodes an AP2 domain-containing transcriptional activator that binds to the C-repeat/DRE, a cis-acting DNA regulatory element that stimulates transcription in response to low temperature and water deficit Proc Natl Acad Sci USA 1997 94 1035 40 10.1073/pnas.94.3.1035 9023378 PMC19635

[B40] Harmer S Hogenesch J Straume M Chang H Han B Zhu T Wang X Kreps J Kay S Orchestrated transcription of key pathways in Arabidopsis by the circadian clock Science 2000 290 2110 3 10.1126/science.290.5499.2110 11118138

[B41] Ulmasov T Hagen G Guilfoyle T The ocs element in the soybean GH2/4 promoter is activated by both active and inactive auxin and salicylic acid analogues Plant Mol Biol 1994 26 1055 64 10.1007/BF00040688 7811965

[B42] Ulmasov T Ohmiya A Hagen G Guilfoyle T The soybean GH2/4 gene that encodes a glutathione S-transferase has a promoter that is activated by a wide range of chemical agents Plant Physiol 1995 108 919 27 10.1104/pp.108.3.919 7630972 PMC157441

[B43] Guiltinan M WR JM Quatrano R A plant leucine zipper protein that recognizes an abscisic acid response element Science 1990 250 267 71 10.1126/science.2145628 2145628

[B44] Yang T Poovaiah B A calmodulin-binding/CGCG box DNA-binding protein family involved in multiple signaling pathways in plants J Biol Chem 2002 277 45049 58 10.1074/jbc.M207941200 12218065

[B45] Horan K Jang C Bailey-Serres J Mittler R Shelton C Harper J Zhu J Cushman J Gollery M Girke T Annotating genes of known and unknown function by large-scale coexpression analysis Plant Physiol 2008 147 41 57 10.1104/pp.108.117366 18354039 PMC2330292

[B46] Vandepoele K Quimbaya M Casneuf T Veylder LD de Peer YV Unraveling transcriptional control in arabidopsis using cis-regulatory elements and coexpression networks Plant Physiol 2009 150 535 546 10.1104/pp.109.136028 19357200 PMC2689962

[B47] Dongen SV Graph clustering via a discrete uncoupling process SIAM J Matrix Anal Appl 2008 30 121 141 10.1137/040608635

[B48] Sponsel V Hedden P Davies P Gibberellin biosynthesis and metabolism Plant Hormones: Biosynthesis Signal Transduction, Action! 2004 Dordrecht: Kluwer 62 98

[B49] Weiss D Ori N Mechanisms of cross talk between gibberellin and other hormones Plant Physiology 2007 144 1240 1246 10.1104/pp.107.100370 17616507 PMC1914132

[B50] Ezcurra I Ellerström M Wycliffe P Stalberg K Rask L Interaction between composite elements in the napA promoter: both the B-box ABA-responsive complex and the RY/G complex are necessary for seed-specific expression Plant Mol Biol 1999 40 699 709 10.1023/A:1006206124512 10480393

[B51] Swarbreck D Wilks C Lamesch P Berardini TZ Garcia-Hernandez M Foerster H Li D Meyer T Muller R Ploetz L Radenbaugh A Singh S Swing V Tissier C Zhang P Huala E The Arabidopsis Information Resource (TAIR): gene structure and function annotation Nucl. Acids Res 2007 36 D1009 D10I4 10.1093/nar/gkm965 17986450 PMC2238962

[B52] Obayashi T Hayashi S Saeki M Ohta H Kinoshita K ATTED-II provides coexpressed gene networks for Arabidopsis Nucleic Acids Res 2009 37 D987 91 10.1093/nar/gkn807 18953027 PMC2686564

[B53] Newman M The structure and function of complex networks SIAM Review 2003 45 167 256 10.1137/S003614450342480

[B54] Gene Ontology Consortium The Gene Ontology (GO) database and informatics resource Nucleic Acids Res 2004 32 D258-61 14681407 10.1093/nar/gkh036 PMC308770

[B55] Benjamini Y Hochberg Y Controlling the false discovery rate: a practical and powerful approach to multiple testing J Roy Statist Soc Ser B 1995 57 289 300

